# E2F6 Impairs Glycolysis and Activates BDH1 Expression Prior to Dilated Cardiomyopathy

**DOI:** 10.1371/journal.pone.0170066

**Published:** 2017-01-13

**Authors:** Jennifer L. Major, Aaraf Dewan, Maysoon Salih, John J. Leddy, Balwant S. Tuana

**Affiliations:** 1 University of Ottawa, Dept. CMM, Ottawa, Ontario, Canada; 2 University of Ottawa Heart Institute, Ottawa, Ontario, Canada; Academia Sinica, TAIWAN

## Abstract

**Rationale:**

The E2F pathway plays a critical role in cardiac growth and development, yet its role in cardiac metabolism remains to be defined. Metabolic changes play important roles in human heart failure and studies imply the ketogenic enzyme β-hydroxybutyrate dehydrogenase I (BDH1) is a potential biomarker.

**Objective:**

To define the role of the E2F pathway in cardiac metabolism and dilated cardiomyopathy (DCM) with a focus on BDH1.

**Methods and Results:**

We previously developed transgenic (Tg) mice expressing the transcriptional repressor, E2F6, to interfere with the E2F/Rb pathway in post-natal myocardium. These Tg mice present with an E2F6 dose dependent DCM and deregulated connexin-43 (CX-43) levels in myocardium. Using the Seahorse platform, a 22% decrease in glycolysis was noted in neonatal cardiomyocytes isolated from E2F6-Tg hearts. This was associated with a 39% reduction in the glucose transporter GLUT4 and 50% less activation of the regulator of glucose metabolism AKT2. The specific reduction of cyclin B1 (70%) in Tg myocardium implicates its importance in supporting glycolysis in the postnatal heart. No changes in cyclin D expression (known to regulate mitochondrial activity) were noted and lipid metabolism remained unchanged in neonatal cardiomyocytes from Tg hearts. However, E2F6 induced a 40-fold increase of the *Bdh1* transcript and 890% increase in its protein levels in hearts from Tg pups implying a potential impact on ketolysis. By contrast, BDH1 expression is not activated until adulthood in normal myocardium. Neonatal cardiomyocytes from Wt hearts incubated with the ketone β-hydroxybutyrate (β-OHB) showed a 100% increase in CX-43 protein levels, implying a role for ketone signaling in gap junction biology. Neonatal cardiomyocyte cultures from Tg hearts exhibited enhanced levels of BDH1 and CX-43 and were not responsive to β-OHB.

**Conclusions:**

The data reveal a novel role for the E2F pathway in regulating glycolysis in the developing myocardium through a mechanism involving cyclin B1. We reveal BDH1 expression as an early biomarker of heart failure and its potential impact, through ketone signaling, on CX-43 levels in E2F6-induced DCM.

## Introduction

The failing heart shows transcriptional and metabolic remodeling which may have detrimental effects on cardiac function[1,2]. Given the extensive energy requirements of the heart, and its limited ATP reserve, understanding the mechanisms which regulate cardiac metabolism is critical to the understanding of heart function and failure [3,4]. In the normal adult heart, fatty acid oxidation accounts for up to 90% of the ATP production while glycolysis supplies the remainder [5]. The heart shows a remarkable capacity to adapt to substrate utilization under stress. In the failing heart, a reduction of fatty acid oxidation and changes in glycolysis are observed [5,6]. It was recently demonstrated that there is an increase in ketone metabolism and the ketogenic enzyme, β-hydroxybutyrate dehydrogenase 1 (BDH1), in human and mouse heart failure [7–10].

Ketones are synthesized in the liver and exist in three main forms: β-hydroxybutyrate (β-OHB) the most stable and abundant form, acetoacetate, and acetone [11]. Ketone bodies arise from the incomplete oxidation of long chain fatty acids. They are utilized as an alternative energy source when glucose is scarce, such as during fasting, exercise, and in many disease states such as diabetes [11, 12]. Glucose is a vital nutrient source in the developing heart and during heart failure, thus ketones could be an important alternative energy source during metabolic stress [1]. It is therefore critical to understand how the heart uses ketones to adapt to substrate utilization during cardiac development and disease.

The E2F family is a group of nine transcription factors which regulate cell proliferation, hypertrophy, and death [13, 14]. Recently, the transcriptional activator, E2F1, has been demonstrated to promote glycolysis via regulation of the pyruvate dehydrogenase kinase (PDK4) and inhibition of histone deacetylases (HDACS) to regulate metabolism [15–18]. In association with its modulator, Retinoblastoma protein (Rb), E2F1 can also repress oxidative phosphorylation[19,20]. E2F6 is a unique E2F family member which is believed to repress E2F responsive genes independently of Rb [21, 22] (Gaubatz, Wood, & Livingston, 1998; Trimarchi, Fairchild, Wen, & Lees, 2001)_._ We previously examined the contribution of the E2F pathway in post-natal mouse myocardium by cardiac specific expression of E2F6 which led to the early-onset of dilated cardiomyopathy (DCM) without hypertrophy or apoptosis [23, 24]. Contrary to expectation, E2F6-Tg mice showed activation of E2F responsive genes [23]. This was achieved via down-regulation of E2F3 (critical for cardiac development) [23, 25]and competitive binding at E2F sites which are normally repressed via E2F/Rb in the post-natal heart[26,27,21]. In essence, E2F6 serves as a dominant negative of E2F/Rb in the heart.

E2F6-Tg mice display an early reduction in connexin-43(CX-43) and reduced conductivity which is a hallmark of DCM and heart failure[25]. Here we further examine the changes in the post-natal myocardium of E2F6-Tg mice with the view to define early bio-markers and novel pathways which may be useful for understanding and treating idiopathic DCM. We note the early induction of BDH1 in neonatal Tg myocardium and a relationship between ketones and CX-43 expression in cardiomyocytes. We further note that deregulation of the E2F pathway impairs glycolysis which may have triggered the induction of BDH1 and the resulting changes in CX-43 leading to DCM.

## Methods

### Mice and Genotyping

Previously described Tg mice with cardiac specific expression of E2F6 (under control of the α-Myosin heavy chain promoter) (B6C3F1) were bred with WT (B6C3F1) mice [23,24]. All animal work was conducted according to the University of Ottawa’s institutional animal care committee guidelines. The protocols were approved by the University of Ottawa's institutional animal care committee: cmm-1725, cmm-1723. All possible steps were taken to ameliorate animal suffering. Mouse pups were euthanized by decapitation. Adult mice were euthanized via carbon dioxide inhalation. Approximately 110 mice were used in this study. This number was required in order to obtain sufficient sample size for appropriate biochemical and statistical analyses.

Genotyping was performed via DNA extraction from mouse ear (adult) or tail (pup) clip and PCR using the Phire Tissue Direct (Thermo Scientific) kit as per the manufacturer’s instructions. Primers spanning the 6^th^ intron of E2F6 were used (ATCACAGTACATATTAGGAGCAC- sense, and GGTGCGGCTACCAGTCTACA–anti-sense) which result in the amplification of a long fragment (988bp) in Wt mice and a long and short fragment (342bp) in Tg mice which were separated on a 1.3% agarose gel.

### RNA Analyses

RNA was extracted from cardiac lysate at post-natal day 7 using the RNEasy Fibrous Tissue Mini Kit as per the manufacturer’s protocol (Qiagen). First-strand cDNA was synthesized from 2μg RNA and oligoDT with SuperScriptII reverse transcriptase (Invitrogen) as per the manufacturer's protocol. qPCR was performed in the q-Rotor (Qiagen) using Fast Start SYBR Green (Roche) according to the manufacturer’s instructions. Gene expression was normalized against *Gapdh*, and fold inductions were calculated using the ΔΔ*C*_*t*_ method. Primer pairs used for qPCR are: *Bdh1*: AAGCACTGGAAGCAGACACAT (sense), ACACTTAGGGCTTTTCCTGGG (anti-sense), *Oxct1*: CTGGAGTTTGAGGACGGCAT (sense), TCCGCATCAGCTTCGTCTTT (anti-sense), *GLUT4*: GCAGATCGGCTCTGACGATG (sense), GCCACGTTGCATTGTAGCTC (anti-sense), and *Gapdh*: GCAGTGGCAAAGTGGAGATT (sense) and TCTCCATGGTGGTGAAGACA (anti-sense).

### Western Blot

Neonatal cardiomyocytes were rinsed twice with PBS and frozen at -80°C. Plates were thawed on ice in RIPA (50mM Tris (pH 7.4), 1mM EDTA, 150μM NaCl, 0.25% deoxycholic acid, 1% NP-40) containing protease and phosphatase inhibitors (Roche). Plates were scraped and lysates were syringed with a 21G needle. Cardiac lysates were prepared with an electric tissue homogenizer in RIPA with protease and phosphatase inhibitors.

Cardiac and cell lysates were centrifuged at 12800g for 10min at 4°C. Protein concentrations were determined using the BCA assay (Thermo Scientific). Lysates (13-50ug) were run on gradient (5–15%) SDS-PAGE gels in 3X loading dye (Cell Signaling). Gels were transferred to PVDF membrane (Millipore) in transfer buffer (25mM Tris, 190mM Glycine, 20% methanol) overnight at 4°C. Membranes were blocked in TBST (1M Tris, 290mM NaCl, 0.1% Tween, pH7.2) with 5% milk. The blots were probed with primary and secondary antibodies (listed below) which were diluted in TBST with 5% milk. The conversion of ECL substrate (Roche) was detected on film. Band signal intensities were quantified by densitometry using Image Lab Software4.0.1 (Bio-Rad).

The following primary antibodies were used: GLUT4 (2213, 1:1000), AKT2 (6063, 1:1000), p-AKT2 (ser 479) (8599, 1:1000), cyclin D1 (2978, 1:1000), cyclin D3 (2936, 1:2000), and cyclin B1 (4138, 1:1000) were purchased from Cell Signaling, BDH1 (MA5-15594, 1:1000) and GAPDH (MA5-15738, 1:5000) were purchased from Thermo Scientific, CX-43 (ab11370, 1:100000) was purchased from Abcam, OXCT1 (12175-1-AP, 1:10000) was purchased from Proteintech, and myc (which detects myc-tagged E2F6) (11667149001, 1:2000) was purchased from Roche. Secondary antibodies anti-mouse (115-035-003, 1:15000) and anti-rabbit (111-035-045, 1:15000) were purchased from Jackson Immunochemicals.

### Neonatal Cardiomyocyte Isolation and Treatment

Hearts were collected from Wt and Tg mice at post-natal day 1 following decapitation. Hearts were rinsed in HBSS and incubated in 0.5% trypsin dissolved in HBSS overnight at 4°C while genotyping was performed. Hearts were grouped in twos or threes (by genotype) for digestion with 0.5% Collagenase type II (Gibco) dissolved in HBSS (4x10min). Cells were collected after each digestion via centrifugation at 3000g for 3 minutes and resuspended in DMEM supplemented with 10% FBS, 1% penicillin/streptomycin, and 1% non-essential amino acids. Total suspensions were plated on uncoated 10cm dishes to remove fibroblasts (45min x2). Cardiomyocytes were seeded onto 0.1% gelatin coated plates (300000/well on XF^e^24 well plate for Seahorse metabolic analysis, or 1x10^6^/well on 6 well plates for ketone treatment) and allowed to attach for 48 hours in a 37°C incubator with 5% CO_2_.

Paired neonatal cardiomyocytes were treated with or without 3.8mM β-hydroxybutyrate (Sigma) dissolved in water for 24 hours, or used for glycolysis and fatty acid oxidation measurements (described below).

### Seahorse Glycolysis Measurement

Cardiomyocyte media was replaced with bicarbonate and glucose free DMEM containing 2mM glutamine. The extracellular acidification rate (ECAR) was measured using an XF^e^24 Extracellular Flux Analyzer (Seahorse Bioscience). Triplicate pre-injection readings were taken to establish a baseline, followed by triplicate reading after sequential treatment with 20mM glucose, 2mg/mL oligomycin, and 100mM 2-deoxy-glucose. ECAR was corrected to baseline pre-injection measurements.

### Seahorse Fatty Acid Oxidation Measurement

Cardiomyocytes were starved for 24hr in substrate limited medium (DMEM, 0.5mM glucose, 1.0mM Glutamine, 0.5mM L-carnitine, 1% FBS) and replaced with fatty acid oxidation assay medium (111mM NaCl, 4.7mM KCl, 1.25mM CaCl_2_, 2.0mM MgSO_4_, NaH_2_PO_4_, 2.5mM glucose, 0.5mM L-carnitine, and 5mM HEPES). Cardiomyocytes were treated with or without 40μM etomoxir for 15min. The oxygen consumption rate (OCR) was measured using the XF^e^24 Extracellular Flux Analyzer (Seahorse Bioscience). Triplicate pre-injection readings were recorded to establish baseline and cardiomyocytes were sequentially treated with either 17μM BSA or 100 μM palmitate conjugated to BSA and: 2mg/mL oligomycin, 1μM Carbonyl cyanide-4-trifluoromethoxy phenylhydrazone, and 1μM Antimycin-A. OCR was corrected to baseline pre-injection measurements.

### Statistical Analyses

All data were analyzed with a student t-test with the exception of the protein expression data, which was analyzed with a two way mixed ANOVA with repeated measures where indicated. The level of significance was set at *P*<0.05 in all cases.

## Results

### E2F6 Induces the Early Expression of BDH1 in Neonatal Myocardium

**Fig 1:**
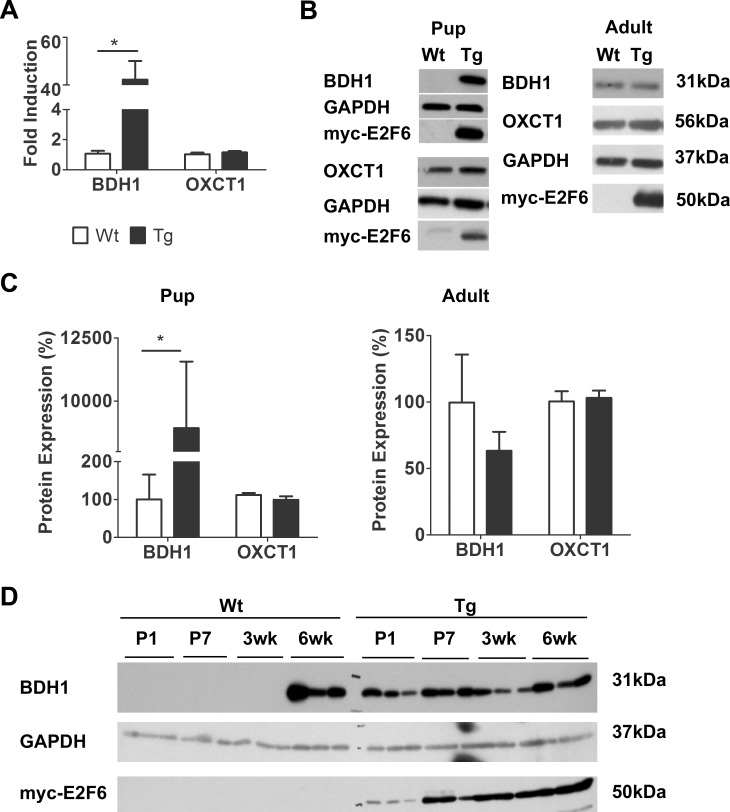
E2F6 Activates BDH1 Expression in Neonatal Myocardium. (**A)** *Bdh1* (β-hydroxybutyrate dehydrogenase 1) and *Oxct1* (succinyl-CoA: 3-oxoacid-CoA transferase) transcript levels in Wt and Tg myocardium 7 days after birth. Expression is normalized to *Gapdh*. Results represent mean±SEM values (n = 5–7). (**B)** Representative immunoblots of protein from Wt and Tg mouse myocardium at post-natal day 1 (pups) and 6wks of age (adult) examined with the anti-BDH1 and anti-OXCT1. (**C)** Densitometric quantification of BDH1 and OXCT1 expression from immunoblots. Expression is normalized against GAPDH. Results represent mean±SEM values (n = 4). (**D)** Immunoblot of protein from Wt and Tg myocardium with BDH1 at the indicated time points after birth.*P<0.05.

E2F6 expression in postnatal myocardium led to dose dependent DCM associated with decreased levels of CX-43[23,24]. Our previous microarray results predicted the up-regulation of *Bdh1* in E2F6-Tg hearts thus we performed RT-q-PCR on mRNA collected from Wt and Tg pup hearts for validation. A 40 fold increase of *Bdh1* transcript was detected in Tg myocardium (*P*<0.05) (Fig 1A). BDH1 protein was barely detectable in Wt pup hearts but was markedly upregulated by 890% (*P*<0.05) in E2F6 Tg hearts at post-natal day 1 (P1) (Fig 1B, quantified in 1C). Since BDH1 is up-regulated in the failing adult human heart [10] we examined its expression at 6 weeks of age, at which time Tg mice show full onset of DCM[24]. Western blot analysis did not reveal any difference in BDH1 level between Tg and Wt hearts at this time (Fig 1B, quantified in 1C).

We next examined the developmental expression of BDH1 protein in post-natal myocardium between P1 and 6wks. In normal developing myocardium BDH1 protein is undetectable at P1 and appears at 6 weeks of age (Fig 1D). On the other hand, in E2F6-Tg hearts, BDH1 expression appears to be maximally expressed as early as P1 with this level maintained at P7, 3 weeks, and 6 weeks. It should be noted that symptoms of DCM arise as early as 2 weeks in E2F6-Tg mice[24], thus BDH1 is markedly elevated prior to the early development of DCM and maintained at this level into adulthood (Fig 1D).

The induction of BDH1 expression by E2F6 was specific since the succinyl CoA: 3-oxoacid CoA transferase (OXCT1) (another rate limiting enzyme in ketone metabolism) was not changed at the mRNA (Fig 1A) or protein level in heart tissue from pup or adult E2F6-Tg mice (Fig 1B, quantified 1C).

### E2F6 Impairs Glycolysis in Neonatal Cardiomyocytes

**Fig 2:**
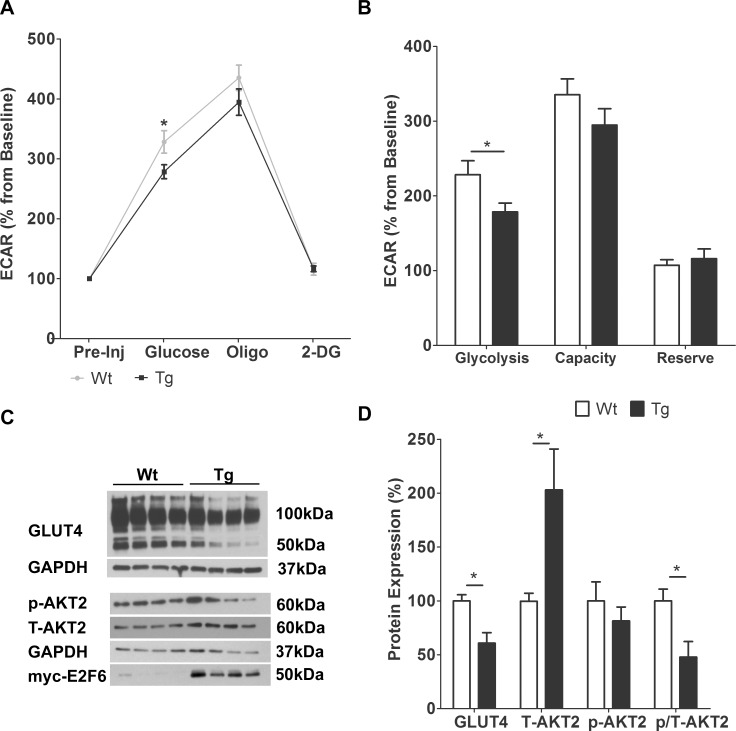
E2F6 Impairs Glycolysis in Neonatal Cardiomyocytes. (**A)** Glycolytic rates for Wt and Tg neonatal cardiomyocytes pre-injection, and following the sequential addition of: glucose, oligo (oligomycin), and 2-DG (2-deoxy glucose). ECAR (extracellular acidification rate) is normalized against pre-injection rates. Results represent mean±SEM values (n = 8). (**B)** Glycolytic measurements (basal glycolysis, glycolytic capacity, glycolytic reserve) in Wt and Tg neonatal cardiomyocytes. (**C)** Representative immunoblot analyses of protein extracts from Wt and Tg myocardium at postnatal day 1 with anti: GLUT 4 (glucose transporter 4), T-AKT2 (total protein kinase B), and p-AKT2 (phospho (ser 474)- protein kinase B). (**D)** Densitometric quantification of immunoblots. Expression is normalized against GAPDH (n = 4). *P<0.*05*.

Since ketone metabolism is increased when glucose is scarce, we measured glycolysis in in Tg mice to gauge whether changes in glycolytic rates may have stimulated BDH1 expression. We used the Seahorse method to monitor the extracellular acidification rate (ECAR) as a measure of glycolysis which produces lactic acid. In nutrient starved neonatal cardiomyocytes (pre-injection) ECAR measurements were similar in Wt and Tg, but Tg ECAR readings were lower following the addition of glucose and oligomycin (inhibitor of ATP synthase that drives glycolysis) (Fig 2A). The competitive inhibitor of glucose, 2-Deoxy-glucose (2-DG), caused the ECAR readings to return to baseline in both Wt and Tg cardiomyocytes demonstrating the specificity of the experiment to glucose (Fig 2A). Calculation of basal glycolysis revealed a 22% decrease in E2F6 expressing cardiomyocytes (*P*<0.05) (Fig 2B). No significant changes in glycolytic capacity or reserve were noted.

Glucose entry into cardiomyocytes is a rate limiting step in glycolysis, thus we examined changes in the cardiac glucose transporter: GLUT4.Western blot analysis of protein isolated from Wt and Tg myocardium at P1 with anti-GLUT4 detected a GLUT4 monomer of ~50kDa and high molecular weight polypeptides which are GLUT4 oligomers (Fig 2C). Quantification of all polypeptides revealed a 39% decrease in total GLUT4 protein (*P*<0.05) (Fig 2D). RT-q-PCR revealed no change in *GLUT4* transcript levels in Tg myocardium (S1 Fig) indicating its loss is post-transcriptional.

We also examined the expression of another major regulator of glucose metabolism: AKT2 (also known as protein kinase B). Western blot analysis with anti- total AKT2 (T-AKT2) revealed a 100% increase in E2F6-Tg hearts (*P*<0.05), while analysis of phosphorylated (active) AKT2 revealed no change (Fig 2C, quantified in Fig 2D). The ratio of phospho: total AKT2 was decreased by ~50% (*P*<0.05) in Tg myocardium indicating less AKT2 activation as a fraction of the total AKT2 pool (Fig 2D).

### E2F6 Does Not Impact Fatty Acid Oxidation in Neonatal Cardiomyocytes

**Fig 3:**
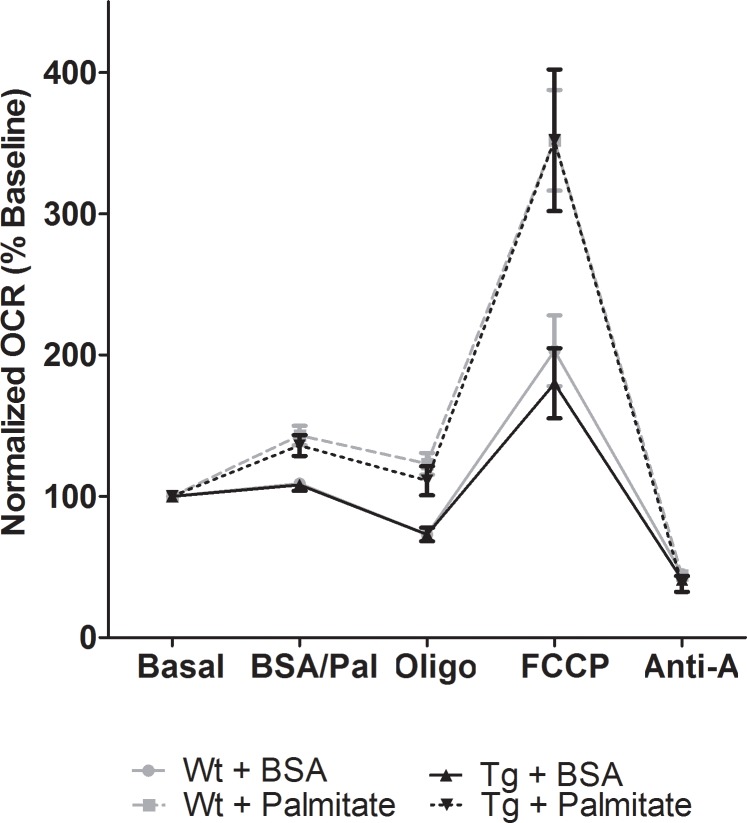
E2F6 Does Not Impact Fatty Acid Oxidation in Neonatal Cardiomyocytes. Oxygen consumption rate (OCR) in Wt and Tg neonatal cardiomyocytes following 24hr glucose starvation. Results were normalized against baseline (pre-injection). Cardiomyocytes were sequentially incubated with BSA or palmitate, oligo (oligomycin), FCCP (Carbonyl cyanide-4-phenylhydrazone), and Anti-A (Antimycin-A). Results represent mean±SEM values (n = 7–8).

In the failing human heart the up-regulation of BDH1 is believed to be an adaptation to a decrease in lipid metabolism[8,9]. Thus, we also assessed lipid metabolism in neonatal cardiomyocytes from Wt and Tg mice. We used the seahorse method to measure the oxygen consumption rate (OCR) which revealed no change in fatty acid oxidation in E2F6-expressing cardiomyocytes at pre-injection, or following the sequential addition of palmitate (fatty acid), oligomycin (inhibitor of ATP synthase), Carbonyl cyanide-4-phenylhydrazone (FCCP/uncoupler) or antimycin-A (electron transport chain inhibitor) (Fig 3). Cardiomyocytes treated with etomoxir (inhibitor of fatty acid entry) showed no significant increase in OCR beyond baseline, thereby demonstrating the specificity of the experiments to fatty acids (S2 Fig).

### E2F6 Deregulates Cyclin Expression in Myocardium

**Fig 4:**
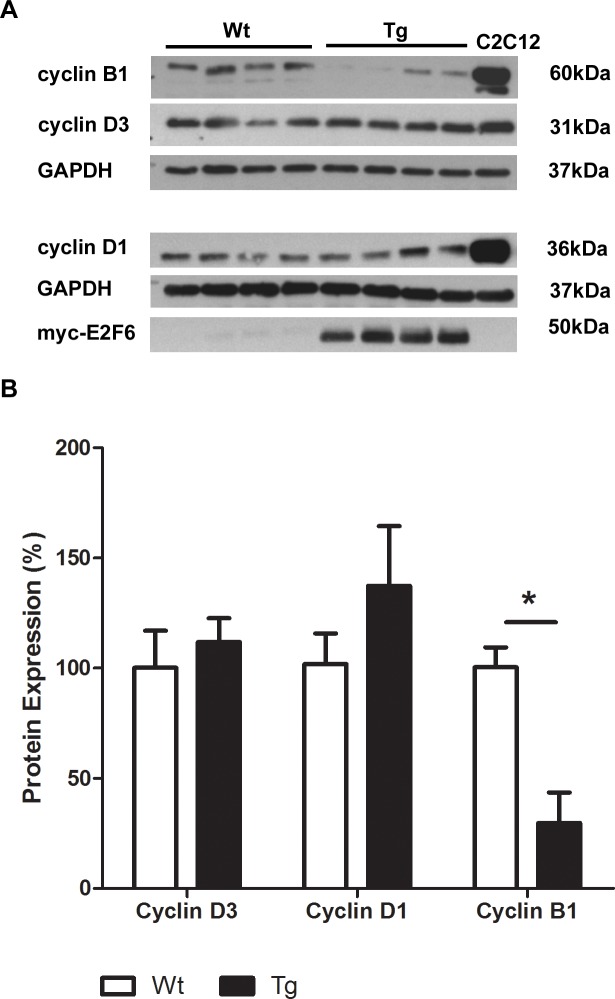
E2F6 Deregulates Cyclin B1 Expression in Myocardium. (**A)** Representative immunoblot of protein isolated from Wt and Tg myocardium at post-natal day 1 with anti- cyclin D1, anti-cyclin D3, and anti-cyclin B1. (**B)** Densitometric quantification of cyclin immunoblots. Expression is normalized against GAPDH. Results represent mean±SEM values (n = 4). *P<0.*05*.

The E2F/Rb pathway is believed to drive glycolysis via the transcriptional regulation of cyclins [28]. Thus, we examined the expression of various cyclins in Wt and Tg myocardium at post-natal day 1. Cyclin D1 and D3 expression levels were not altered in cardiac tissue from E2F6-Tg mice (Fig 4A, quantified in 4B) while cyclin B1 was reduced by 70% in E2F6-Tg myocardium (*P*<0.05) (Fig 4A, quantified in 4B).

### Ketones Regulate Connexin-43 in Neonatal Cardiomyocytes

**Fig 5:**
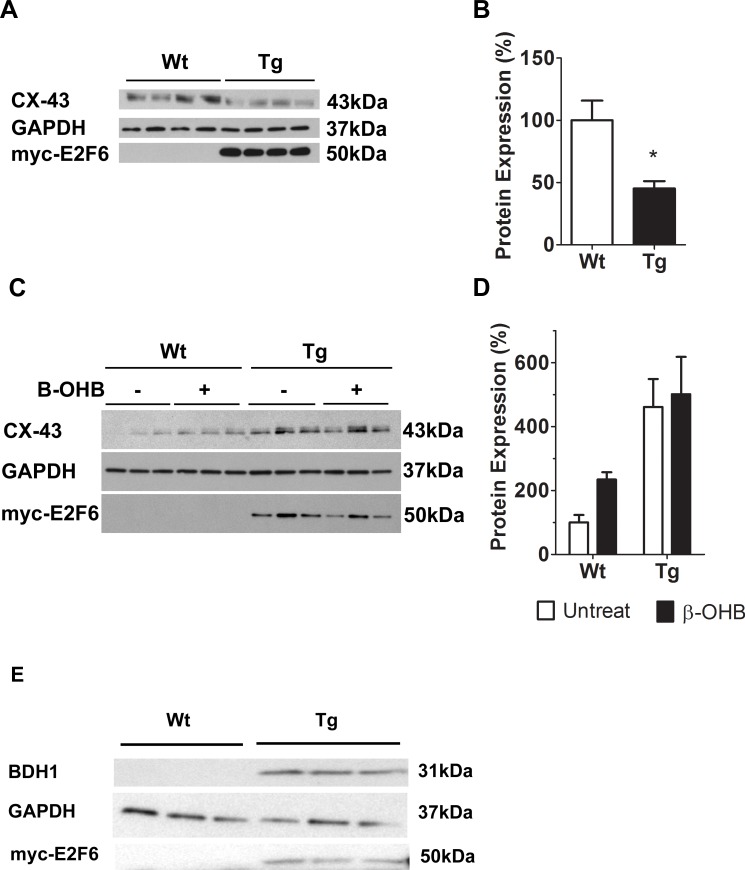
β-OHB Regulates CX-43 Protein Expression in Neonatal Cardiomyocytes. (**A)** Representative immunoblot of protein isolated from Wt and Tg myocardium at post-natal day 1 with anti-CX-43. (**B)** Densitometric quantification of CX-43 immunoblot. Expression is normalized against GAPDH. Results represent mean±SEM values (n = 4). (**C)** Representative immunoblot analyses of protein from Wt and Tg neonatal cardiomyocytes following incubation with or without β-OHB (β-hydroxybutyrate). (**D)** Densitometric quantification of CX-43 in treated and non-treated neonatal cardiomyocytes. Expression is normalized against GAPDH. Results represent the mean±SEM values (n = 3–5). A two-way ANOVA was used to measure statistical significance. (**E**) Representative immunoblot analysis of BDH1 in Wt and Tg neonatal cardiomyocytes. *P<0.*05*.

Recent evidence indicates that ketones can enhance CX-43 protein in endothelial cells via the Extracellular Receptor Kinase (ERK) pathway[29]. We previously noted the deregulation of ERK activity and a loss of CX-43 protein in adult Tg mice[23], and in the present study we detected a 50% down-regulation of CX-43 protein at post-natal day 1 (*P*<0.05) (Fig 5A, quantified in 5B).

We hypothesized that the excess BDH1present in Tg hearts could limit the amount of β-OHB in the cardiomyocyte via its conversion to acetoacetate and subsequent availability for the ketone catabolic pathway. This could in turn inhibit the signaling capacity of β-OHB (the most stable and abundant ketone) and thus may contribute to the observed down-regulation of CX-43 in Tg hearts. To test this we examined the amount of CX-43 in neonatal cardiomyocytes incubated with or without the β-OHB. Wt cardiomyocytes incubated with β-OHB showed a 100% increase of CX-43 (*P*<0.05) (Fig 5C, quantified in 5D), similar to what was recently reported in endothelial cells[29]. In contrast, β-OHB had no significant impact on CX-43 expression in Tg cardiomyocytes supporting our hypothesis that BDH1 reduces ketone signaling.

Surprisingly, the reduction of CX-43 noted *in vivo* (Fig 5A) was reversed in culture of Tg cardiomyocytes (Fig 5B). In order to rule out the possibility that BDH1 was reduced in culture, thereby reversing its effect on CX-43 expression, we assessed its level in cultured cardiomyocytes. Western blot with anti-BDH1 detected its presence in neonatal cardiomyocytes cultured from Tg mice, but not Wt, indicating that its loss is not responsible for the rescue of CX-43 in culture (Fig 5E). This suggests the presence of factors in the culture media which specifically promote expression of CX-43 in neonatal cardiomyocyte from Tg hearts. In support of this hypothesis, Tg cardiomyocytes showed an increase in CX-43 expression over time in culture (S4 Fig).

## Discussion

The E2F pathway regulates early cardiac development through mechanisms that impact cell proliferation, hypertrophy, and death[30–33]. We previously demonstrated that the E2F pathway can also impact post-natal cardiac function via manipulation of the pathway *in vivo* with the repressor E2F6[23,24]. E2F6 resulted in dose dependent DCM associated with inappropriate activation of E2F responsive genes, but without changes in cardiac growth or death[23,24]. Further, our data also revealed that the E2F pathway can impact β-adrenergic signaling in the post-natal heart[24]. Here we demonstrate that deregulation of the E2F pathway impairs glycolysis in the developing postnatal myocardium, and markedly enhances that expression of the ketogenic enzyme, BDH1, which was linked to the down-regulation of CX-43. Thus we present notable evidence that appropriate modulation of the E2F pathway is critical for regulating cardiac metabolism and, consequently, function of post-natal myocardium.

E2F6-Tg mice present with an early reduction of CX-43 and a defect in cardiac function potentially accounting for the noted DCM and sudden death [23]. Recent studies demonstrated that β–OHB can enhance CX-43 expression in endothelial cells [29] which we have now also noted in Wt neonatal cardiomyocytes. This suggests that ketones may uniquely modulate the gap junction levels in different cell types. It should be noted that CX-43 expression was rescued in culture of Tg cardiomyocytes while BDH1 was still highly expressed. Thus other factors appear to have an effect on CX-43 expression in culture conditions. In this regard, multiple MAPK pathways have been implicated in the post-transcriptional regulation of CX-43, and these were deregulated in E2F6-Tg mice [34, 29, 23]. It is possible that deregulation of MAPKs may have sensitized E2F6-Tg cardiomyocytes to growth factors in the culture media.

Despite differences in vitro and in vivo both neonatal cardiomyocytes and hearts from Tg mice show a marked induction of BDH1 and a diminished capacity to regulate CX-43 expression. In the heart, Tg-mice do not enhance CX-43 levels to the appropriate level required for cardiac function, and in culture, Tg cardiomyocytes are unresponsive to CX-43 ketone signaling pathways. In both cases this likely involves the marked induction of BDH1 protein which could diminish the available β-OHB (via its conversion to a less stable ketone) thereby driving it towards the ketone catabolic pathway and reducing its ability to act as a signaling molecule to enhance the amount of CX-43. The resulting down-regulation of CX-43 in Tg myocardium would alter cardiomyocyte communication and function from a very young age, potentially leading to the early DCM noted. In this regard, CX-43 is reported to be down-regulated in the diabetic rat heart and in many types of heart failure [34–37]. Further, metabolic remodeling is known to occur in heart failure with enhanced BDH1 and ketone signaling [9,10]. Hence our data here may critically serve to link enhanced BDH1 and the loss of CX-43 in the adult failing heart as well. It has previously been demonstrated that an increase in BDH1 transcript levels in both mouse and human cardiac tissue is associated with enhanced BDH1 activity and ketone metabolism [9,10]. While we have not measured BDH1 activity here, the robust increase in both transcript and protein levels induced by E2F6 imply an enhanced BDH1enzyme activity as early as postnatal day 1.

The relationship between E2F, BDH1, and CX-43 highlights the emerging idea that ketones not only serve as energy substrates, but are also important signaling molecules which can regulate gene expression during nutrient scarcity/adaptation. Ketones can initiate undefined signaling cascades via binding to G-protein coupled receptors, or by direct interaction with and inhibition of HDACs [38, 39]_._ Rb recruits HDACs to repress E2F responsive genes [40,41], which we have shown to be inhibited by E2F6 expression in the heart[23]. Furthermore, we found that E2F6 also regulates G-coupled protein receptor signaling pathways [24], suggesting a novel relationship between E2F/Rb, HDACs, and ketones which critically impacts myocardial growth, function, and metabolism.

It is plausible that BDH1 was induced by E2F6 in Tg myocardium to compensate for the impairment in glycolysis (which is the major source of energy in the neonatal heart). The decrease in GLUT4 protein could have limited glucose entry to E2F6-Tg cardiomyocytes, and when coupled with the observed decrease in AKT2 activation (which regulates glucose homeostasis) could account for the impaired glycolysis [42]. Alterations in glucose metabolism have been previously linked to the E2F/Rb pathway as demonstrated by double knock-out of E2F1/E2F2 which impaired insulin production and caused diabetes [43]. Additionally, studies have demonstrated that E2F/Rb promote glycolysis over oxidative phosphorylation in muscle and fat tissue via the deregulation of cyclin, in particular cyclin D1 [28,44]. Thus it is a logical extension that E2F6, which inhibits E2F/Rb activity, would also inhibit glycolysis. E2F6-Tg mice express less cyclinB1 which could represent a delay in the cardiac cell cycle (i.e. not reaching mitosis) similar to what was observed in E2F6 expressing 3T3 cells which accumulated in S phase[21]. Such a shift in the cell cycle could cause a shift in the metabolic profile as well. The specific change in cyclin B1 may involve the reduction of E2F3 in E2F6-Tg myocardium [23] since it is a critical regulator of the cell cycle during perinatal cardiac development [25]. This suggests that E2F3 may also be a key regulator of cyclin B1 and glycolysis in the heart. Further, since fatty acid oxidation is not increased in response to the depression of glycolysis, ketolysis may be a necessary adaptation as an alternative energy source.

It is interesting to note that BDH1 levels were not increased in the adult (6wk old) Tg-myocardium which are experiencing heart failure. This may reflect the fact that fatty acids are the major energy substrate at this time and fatty acid metabolism appeared to be unaffected in E2F6 Tg mice. This implies that the induction of BDH1 may be an early marker for metabolic stress/changes in the myocardium that precede heart failure. There is a window of overlap between enhanced BDH1 expression and the early symptoms of DCM around 3 weeks supporting the correlative relationship [24]. This may be particularly useful information for ~40% of cases of idiopathic dilated cardiomyopathy and early-onset of disease.

The robust increase of *Bdh1* transcript by E2F6 at birth and the lack of change in other ketogenic enzymes, such as OXCT1, imply a specific role for E2Fs through a transcriptional mechanism. A basic promoter analysis did not detect an E2F consensus binding site in the *Bdh1* promoter (S3 Fig). This does not rule out direct transcriptional regulation as E2F6 has been demonstrated to bind to DNA at non-consensus sites and activate transcription [45,46]. A nuclear-factor (NF)-kappaB site was predicted by the promoter analysis 1938bp upstream of the BDH1 transcription start site (core score 1.00). This is of particular interest because E2Fs can bind to and de-regulate NF-kappaB in the context of inflammation and metabolism [47,48]. Thus it is possible that the deregulation of E2Fs in E2F6-Tg myocardium may have enabled NF-kappaB to flip a metabolic switch activating BDH1 in the neonatal heart. In support of this, E2F1-3 levels normally drop in the adult myocardium [49] when we have demonstrated that BDH1 is normally expressed.

The data here demonstrate a novel role for the E2F pathway in regulating metabolism in post-natal myocardium since its perturbation by E2F6 leads to aberrant glycolysis and the induction of the ketogenic enzyme: BDH1. These changes appear to impact ketogenic signaling to deregulate CX-43 levels and thereby disrupt cardiac function. The potent early induction of BDH1 prior to a discernable cardiac pathology implies that BDH1 is an early biomarker of metabolic stress and DCM. The information here could provide new insight into the early diagnosis and potential treatment options in idiopathic cases of DCM.

## Supporting Information

S1 Fig *GLUT4* Transcription is Not Altered in E2F6-Tg myocardium. *GLUT4* transcript levels from Wt and Tg myocardium 7 days after birth. Expression is normalized to *Gapdh*. Results represent mean±SEM values (n = 5–7).
(TIF)

S2 Fig Seahorse Method of OCR is Specific to Fatty Acids. Normalized oxygen consumption rate (OCR) of Wt and Tg neonatal cardiomyocytes following 24hr glucose starvation and treatment with etomoxir. Cardiomyocytes were treated with either BSA or palmitate, followed by the addition of oligomycin (Oligo), Carbonyl cyanide-4-phenylhydrazone (FCCP), and Antimycin-A (Anti-A). Results represent mean±SEM values (n = 7–8).
(TIF)

S3 Fig Promoter Analysis of *Bdh1*. Analysis was performed using P-Match. The DNA sequence 5000bp upstream of the transcription start site of *Bdh1* was analyzed against the vertebrate core matrix.
(TIF)

S4 Fig CX-43 Expression is Increased in Tg Cardiomyocytes With Time in Culture. (A) Representative immunoblot of CX-43 in neonatal cardiomyocytes from Tg mice at 16, 36, and 72hr post-plating. (B) Quantification of CX-43 immunoblots. Results are presented as the mean ± SEM (n = 3). *P<0.05.
(TIF)
