# Supplementary figures and images for: E2F6 Impairs Glycolysis and Activates BDH1 Expression Prior to Dilated Cardiomyopathy
Source: PLoS One. 2017 Jan 13;12(1):e0170066. doi: 10.1371/journal.pone.0170066 (PMC5234782; doi:10.1371/journal.pone.0170066)

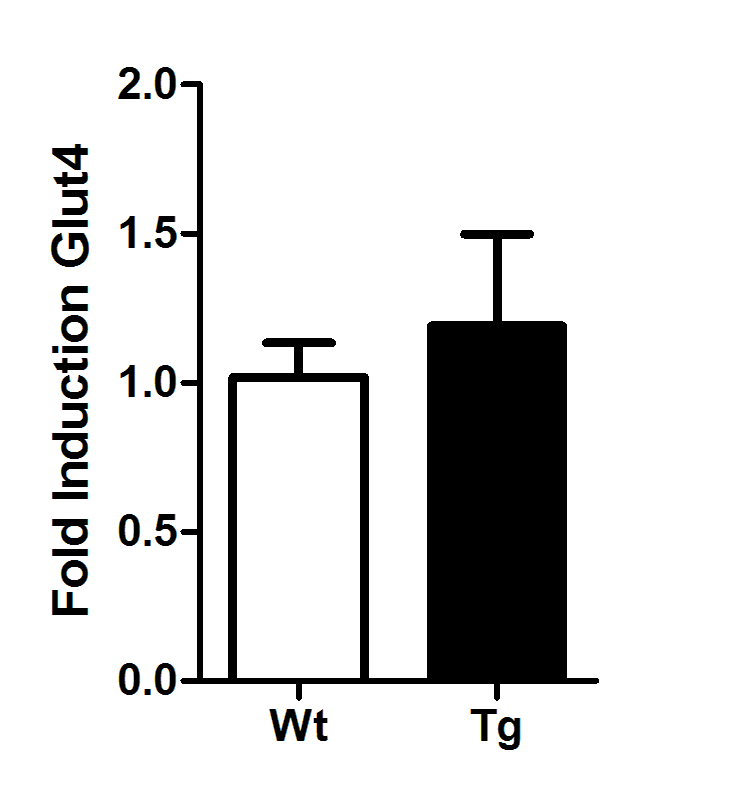

Supplement: S1 Fig — GLUT4 transcript levels from Wt and Tg myocardium 7 days after birth. Expression is normalized to Gapdh. Results represent mean±SEM values (n = 5–7). (TIF) [file pone.0170066.s001.tif]

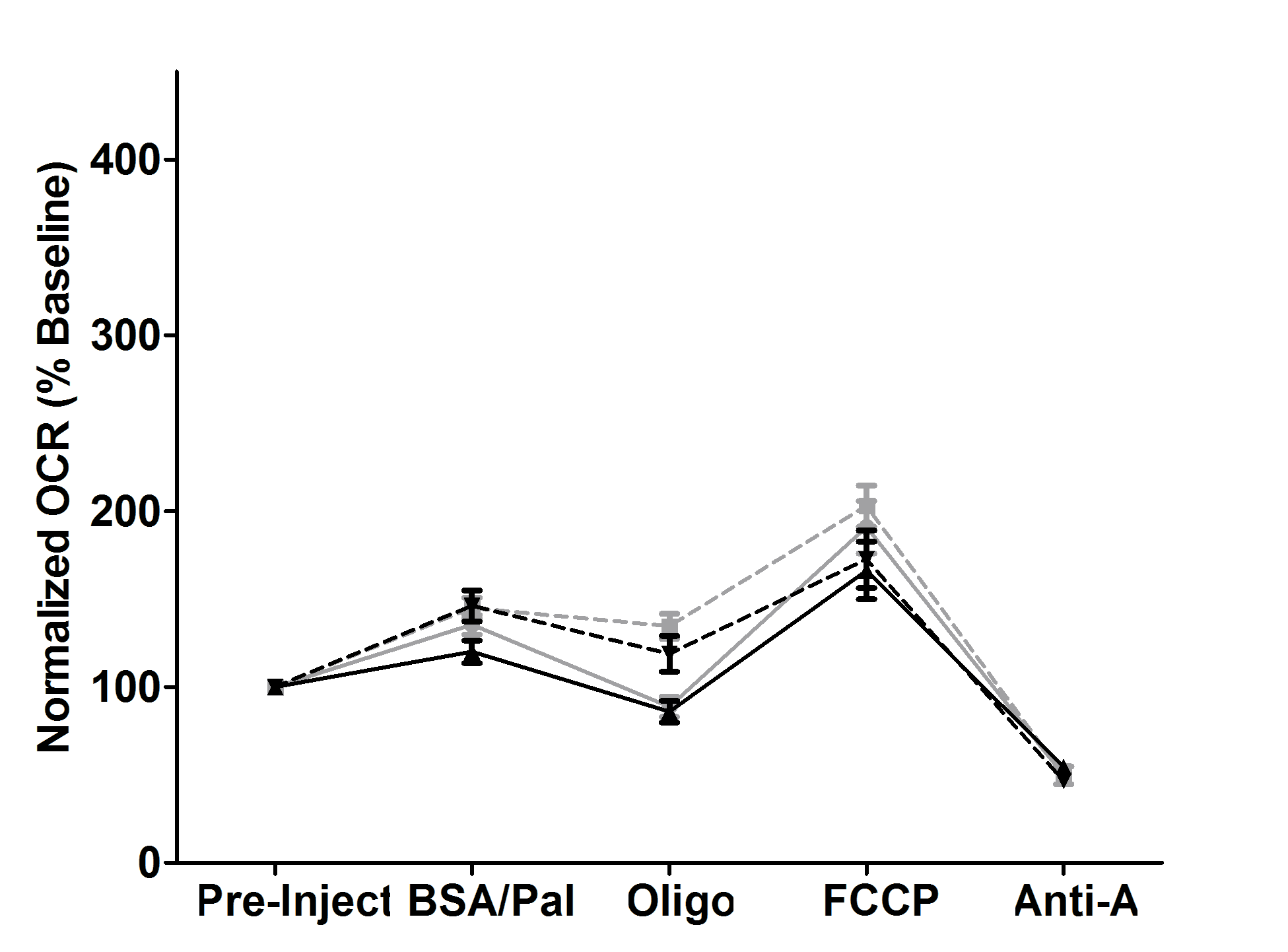

Supplement: S2 Fig — Normalized oxygen consumption rate (OCR) of Wt and Tg neonatal cardiomyocytes following 24hr glucose starvation and treatment with etomoxir. Cardiomyocytes were treated with either BSA or palmitate, followed by the addition of oligomycin (Oligo), Carbonyl cyanide-4-phenylhydrazone (FCCP), and Antimycin-A (Anti-A). Results represent mean±SEM values (n = 7–8). (TIF) [file pone.0170066.s002.tif]

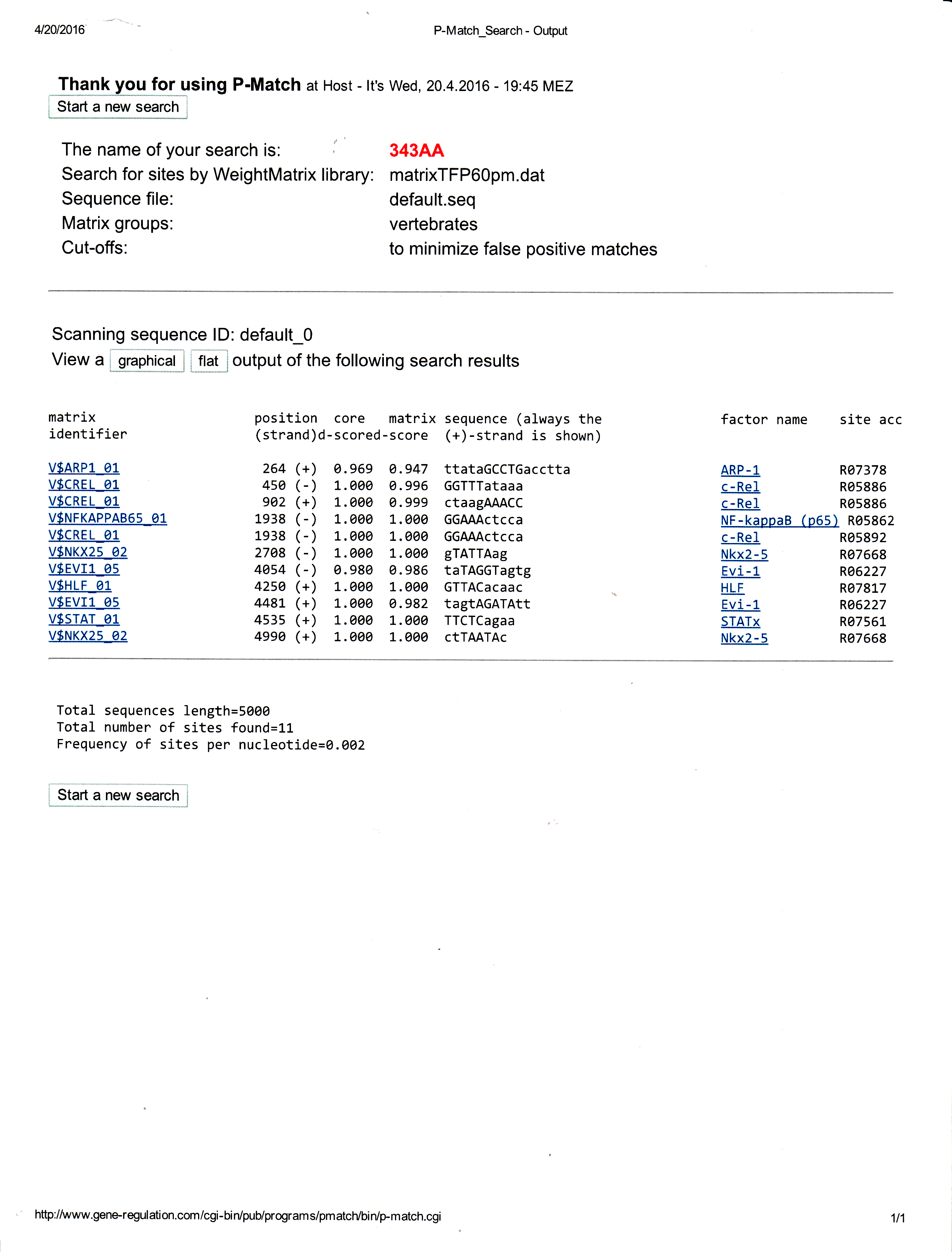

Supplement: S3 Fig — Analysis was performed using P-Match. The DNA sequence 5000bp upstream of the transcription start site of Bdh1 was analyzed against the vertebrate core matrix. (TIF) [file pone.0170066.s003.tif]

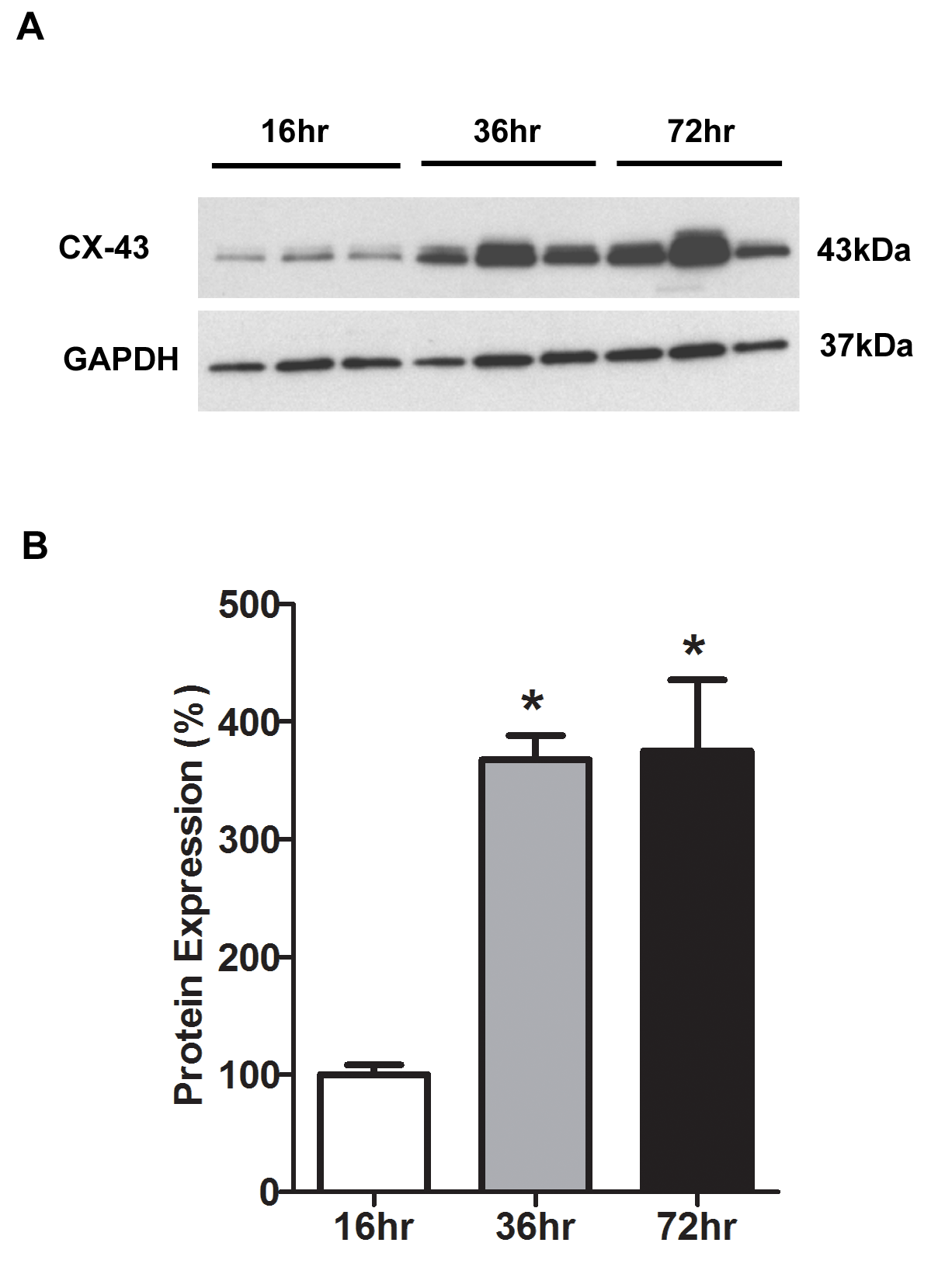

Supplement: S4 Fig — (A) Representative immunoblot of CX-43 in neonatal cardiomyocytes from Tg mice at 16, 36, and 72hr post-plating. (B) Quantification of CX-43 immunoblots. Results are presented as the mean ± SEM (n = 3). *P<0.05. (TIF) [file pone.0170066.s004.tif]
